# Exploring the interplay of gut microbiota, inflammation, and LDL-cholesterol: a multiomics Mendelian randomization analysis of their causal relationship in acute pancreatitis and non-alcoholic fatty liver disease

**DOI:** 10.1186/s12967-024-04996-0

**Published:** 2024-02-19

**Authors:** Congzhi Yan, Jingxia Bao, Jinji Jin

**Affiliations:** 1 Department of Gastrointestinal Surgery, The First Affiliated Hospital of Wenzhou Medical University, Zhejiang, 325000 China; 2 Wenzhou Medical University, Zhejiang, China

**Keywords:** Gut microbiota, Inflammatory cells, Inflammatory proteins, Multiomics

## Abstract

**Background:**

Acute pancreatitis and non-alcoholic fatty liver disease are both serious diseases in the digestive system. The pathogenesis of both diseases is extremely complex closely and it related to gut microbiota, inflammation, and blood fat. There is a close relationship between gut microbiota and blood lipids.

**Methods:**

In this study, we used three types of exposure: 412 gut microbiota, 731 inflammatory cells, and 91 inflammatory proteins (pqtls), with LDL-C as an intermediary and acute pancreatitis and non-alcoholic fatty liver disease as outcomes. We mainly used MR-IVW, co-localization analysis, and reverse MR analysis methods for analysis.

**Results:**

7 gut microbiota, 21 inflammatory cells, and 3 inflammatory proteins can affect LDL-C levels. LDL-C is associated with acute pancreatitis and non-alcoholic fatty liver disease.

**Conclusions:**

Three omics were used: 412 gut microbiota, 731 inflammatory cells, and 91 inflammatory proteins (pqtls). It explains the causal relationship between multiomics, LDL- cholesterol, acute pancreatitis, and non-alcoholic fatty liver disease.

**Supplementary Information:**

The online version contains supplementary material available at 10.1186/s12967-024-04996-0.

## Background

Acute pancreatitis (AP) is marked by an inflammatory cascade triggered by an imbalance in pancreatic cells, often in response to pancreatic digestive enzymes. This imbalanced response induces inflammation, setting off various mechanisms of inflammation progress. In the presence of inflammation and disrupted calcium ion conduction, pancreatitis can initiate a severe systemic inflammatory response, accompanied by extensive self-inflammation within the pancreas [1].

TLR 4, a receptor integral to innate immunity, has emerged as a potential therapeutic target for AP, significantly influencing the development of pancreatic injury during severe acute pancreatitis (SAP) [2, 3]. Moreover, the activation of the NLRP3 inflammasome has been implicated in AP’s pathogenesis. Inhibition of the NLRP3 inflammasome has demonstrated considerable potential in alleviating pancreatic organ damage and systemic inflammation in animal models [4]. Recent investigations have illuminated the role of TLR4 signaling and the NLRP3 inflammasome in hypercholesterolemia and the development of AP. Elevated levels of total cholesterol (TC, > 240 mg/dL) and low-density lipoprotein cholesterol (LDL-C, > 150 mg/dL) within the initial 24 h of hospital admission have been independently link to an increased risk of SAP [5, 6]. Conversely, the occurrence of SAP has been associated with reduced levels of cholesterol-related blood lipids, including total cholesterol, high-density lipoprotein cholesterol (HDL-C), low-density lipoprotein cholesterol, and apolipoprotein A1 [5–7]. As a result, cholesterol-related blood lipids are considered potential risk factors or predictors for SAP onset [5–7]. However, the presence of confounding factors in many studies prevents definitive establishment of a causal relationship between LDL-C and acute pancreatitis. Non-alcoholic fatty liver disease (NAFLD) is a pathological syndrome characterized by hepatic steatosis, denoting the excessive accumulation of lipids in hepatocytes. The etiology of this disease is unrelated to alcohol consumption, but rather associated with other identifiable factors that induce liver injury, including drug use, viral infections, and autoimmune factors [8, 9]. The onset and progression of NAFLD stem from an imbalance in hepatic free fatty acid metabolism Shifts in global dietary patterns and lifestyles have led to a significant rise in the prevalence of overweight and obese individuals, thereby contributing to the increasing incidence of obesity-related chronic diseases such as type 2 diabetes, cardiovascular diseases, metabolic syndrome, and NAFLD [10, 11]. Presently, NAFLD is prevasive worldwide, affecting approximately 25% of the population, with particularly high rates observed in South America and the Middle East [10, 11]. Notably, the prevalence of NAFLD in China has nearly doubled over the past decade, surpassing that of developed countries and reaching an alarming 30% [10]. Projections suggest that by 2030, the global incidence of NAFLD among individuals aged 15 and above will soar to 33.5% [12]. Non-alcoholic fatty liver disease present as a highly heterogeneous condition, intricately influenced by genetic, environmental, and dietary factors [13]. Unhealthy habits further raise circulating levels of fatty acids, promoting excessive transport to the liver. When the liver's capacity for processing fatty acids is exceeded, lipid accumulation occurs, leading to hepatic steatosis and inflammation [14, 15]. Inflammatory processes exacerbate liver cell damage and accelerating the transition from simple fatty liver to NAFLD [16–19]. Furthermore, liver cell death, primarily through apoptosis, plays a significant role in the onset and progression of NAFLD. Comprehensive investigations have revealed that the pathogenesis of NAFLD is tremendously intricate, with the prevailing perspective being the “multiple parallel hits” theory. Various factors, including adipokines, local inflammation, gut microbiota, genetics, and epigenetics, may collectively contribute to the advancement of this disease [19, 20].

Several clinical studies have provided evidence supporting the idea that dyslipidemia acts as an inherent risk factor for non-alcoholic fatty liver disease independently. This is attributed to a distinct correlation between dyslipidemia and severe liver ailments arising from non-alcoholic fatty liver disease, including cirrhosis, cirrhosis-related complications, and liver-related mortality [21]. Research findings indicate that inflammatory responses trigger heightened activity of mammalian target of rapamycin complex 1 (mTORC1), disrupting transcription and post-transcriptional mechanisms, impairing the expression of low-density lipoprotein receptor (LDLR), and exacerbating the progression of non-alcoholic fatty liver disease [22]. Notebly, the gut microbiota shows a significant association with lipid metabolites in the bloodstream [23]. Through the employment of guar gum, the gut microbiota facilitates a reduction in plasma triglycerides (TAG) and LDL-C [24, 25].

It is essential to recognise that previous studies primarily employed case–control study designs, posing challenges in disentangling exposure factors from outcomes. Additionally, confounding factors such as age, environment, dietary patterns, and lifestyle may impact the relationship between gut microbiota, inflammatory cells, inflammatory proteins, and LDL-C in observational studies [26]. These confounding factors can influence the association between LDL-C and non-alcoholic liver diseases, and effectively controlling them in observational studies proves to be demanding. These limitations curtail our ability to draw causal inferences regarding the intricate interplay among gut microbiota, inflammatory cells, inflammatory proteins, and LDL-C in the study of NAFLD.

In this study, a novel approach called Mendelian randomization (MR) was employed to examine the impact of multiple omics on LDL-C levels and to explore the causal relationship between LDL-C and acute pancreatitis, as well as LDL-C and NAFLD. Mendelian randomization utilizes genetic variations as instrumental variables for exposure, enabling the assessment of causal associations between exposure and disease outcomes [27]. SMR is a process that assesses genes whose levels of expression may be causally linked to an outcome variable by integrating summary statistics from GWAS and eQTL studies under the MR paradigm. Genetic variations are instrumental variables in MR analysis that are used for establishing a link of causation between an exposure and an outcome. As the distribution of genotypes from parents to offspring is random, the association between genetic variations and outcomes remains unaffected by common confounding factors. MR has been widely deployed to investigate causal relationships in various diseases. In this particular study, a comprehensive genome-wide association study (GWAS) involving 412 gut microbiota, inflammatory cells, and inflammatory proteins was conducted to investigate the causal relationship between NAFLD, and acute pancreatitis. A two-step Mendelian randomization model was implemented to ascertain whether LDL-C acts as a mediator.

## Materials and methods

### Data of gut microbiota, plasma pQTL and immune cells

**Fig. 1:**
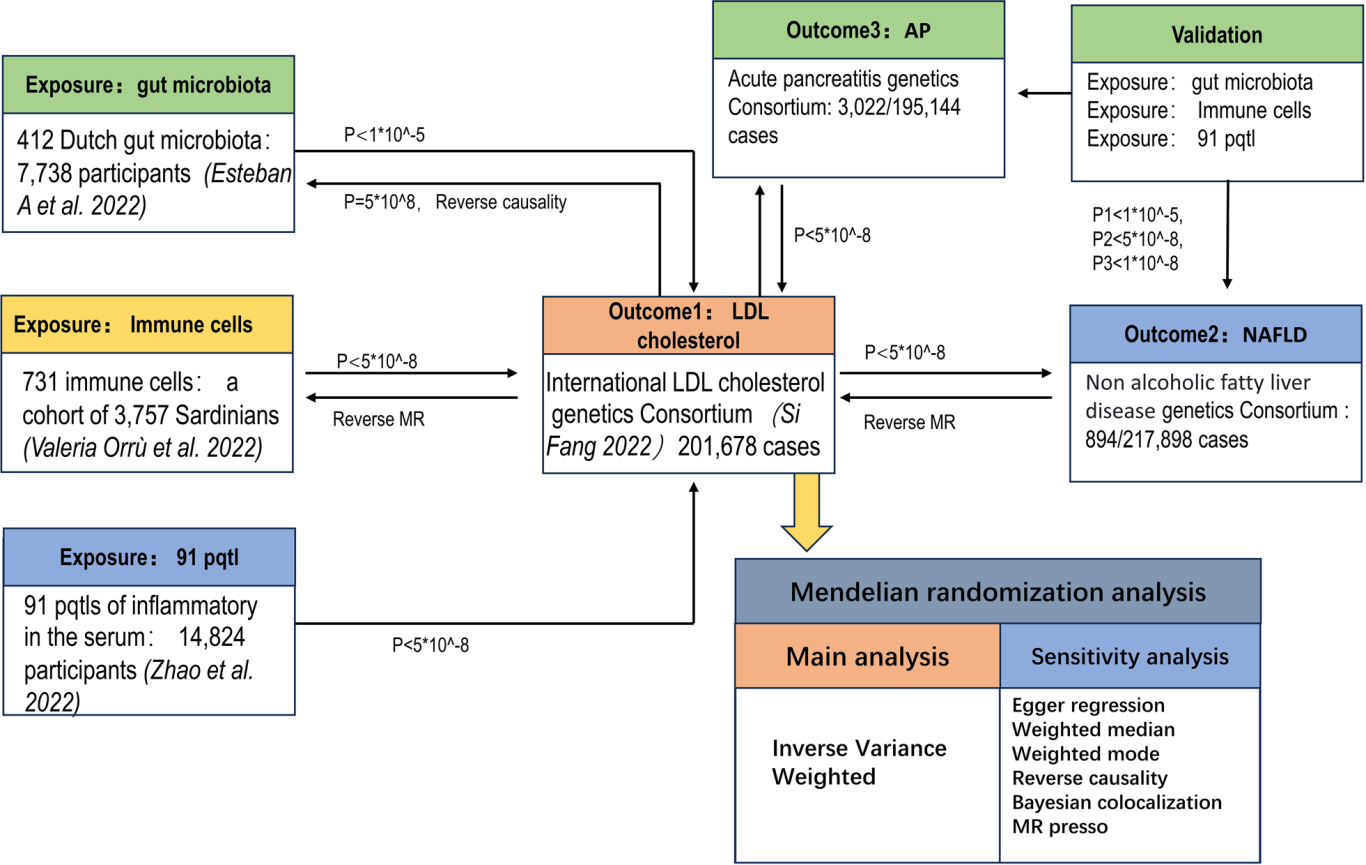
The workflow of this study

Gut microbiota data were obtained from a study conducted by Esteban et al. [28], who reported 412 microbiotas in the gut. 7738 people participated in the study, which carried out a genome-wide association study involving 207 taxa and 205 pathways that reflect the composition and activity of microorganisms. Only microbiotas’ genetic instruments that met the following criteria were included: (a) exhibited genome-wide significant association (*P* < 1 × 10^−5^), (b) demonstrated independent association linkage disequilibrium (LD) clumping *r*^*2*^ < 0.001 and kb < 10,000, (c) minor allele frequency (MAF) > 0.01. For the data of immune cells, the Single Nucleotide Polymorphism (SNP)data were retrieved from the study conducted by Valeria Orrù et al. [29]. The criteria for the genetic instruments were as follows: *P* < 5 × 10^−8^, r^2^ < 0.001, MAF > 0.01 and kb < 10,000. Additionally, the plasma pQTL data were obtained from a recently published study by Zhao et al. [30]. The criteria of genetic instruments was: *P* < 5 × 10^−8^, r^2^ < 0.001, MAF > 0.01 and kb < 10,000. The Fig. 1 shows the workflow in this study.

### GWAS summary statistics of LDL-C and non-alcoholic fatty liver disease

The LDL-C data were acquired from the open GWAS published study by Si Fang et al. (https://gwas.mrcieu.ac.uk/), encompassing a total of 201,678 individuals (ID: ieu-b-5089). When LDL-C was used as exposure and its criteria for the LDL-C genetic instruments were as follows: *P* < 5 × 10^−8^, *r*^*2*^ < 0.001, MAF > 0.01 and kb < 10,000. The acute pancreatitis data were extracted from the open GWAS (https://gwas.mrcieu.ac.uk/), specifically from the FinnGen study, which consisted of 3,022 cases and 195,144 controls. When acute pancreatitis data was used as exposure and its criteria for the genetic instruments were as follows: P < 5 × 10^−8^, r2 < 0.001, MAF > 0.01 and kb < 10,000. The Non-alcoholic fatty liver disease data were extracted from the open GWAS, specifically from the FinnGen study, which consisted of 894 cases and 217,898 controls. When Non-alcoholic fatty liver disease data was used as exposure and its criteria for the genetic instruments were as follows: *P* < 5 × 10^−8^, *r*^*2*^ < 0.001, MAF > 0.01 and kb < 10,000. All the above data were obtained from relevant literature and public databases, with participant consent and ethical review. Therefore, this study of ethical approval from the institutional review board are not necessary. The F statistic, F = R^2^(n − 2)/(1 − R^2^), is used to measure the strength of each genetic tool, where N is the effective sample size for GWAS for SNP association.

### Mendelian randomization analysis

In this study, we first used 412 gut microbiota, 731 inflammatory cells, and 91 inflammatory proteins as exposure factors respectively, with LDL-C as the outcome measure, and conducted analyze using MR with the ‘TwoSampleMR’ package. Inverse-variance weighted (IVW) was used in the analysis as a mainly method to assess causal relationships between exposures and outcomes. Next, we used LDL-C as the exposure factor and NAFLD as the outcome measure, and similarly analyzed the causal relationship between the two using the ‘TwoSampleMR’ package in MR. Inverse variance weighted MR (MR-IVW), and inverse variance weighted- fixed effects MR (MR-IVW-FE) were mainly analysis methods. Egger regression (MR-Egger), weighted median, weighted mode, Bayesian colocalization, and MR-Presso were applied in the analyze. IVW is mainly used to assess the causal relationship between exposure factors and outcome factors. When the statistical P value of MR-Egger is less than 0.05, it can be considered that there is heterogeneity between exposure and outcome. Weighted median, and weighted mode were used to assess horizontal pleiotropy between exposure and outcome. The effect size and associated 95% confidence interval were estimated using the inverse variance-weighted fixed-effect approach. The SMR technique was created to investigate the pleiotropic relationships that exist between significant complex factors like illness phenotype and genetic traits like gene expression, DNA methylation, or protein abundance. Causality was evaluated using the following formula to make sure the Mendelian randomization (MR) principles were followed in our analysis: βpQTL − LDL-C = βSNP − LDL-C/βSNP − pQTL. The estimated influence of SNP on the genes or traits that are determined by those genes is represented by βSNP-eQTL (i.e., genetic variation-exposed trait association), and the estimated effect size of SNP on LDL-C (i.e., the same genetic variation-outcome trait association) is represented by βSNP-LDL-C.

### Reverse causality detection

According to the same selection criteria above, we selected SNP that met the criteria from genome-wide association studies on acute pancreatitis, and non-alcoholic fatty liver disease, and LDL-C for bidirectional Mendelian randomization analysis to explore potential reverse causal relationships. We obtained corresponding comprehensive summary statistics data from previous studies and conducted analyses using methods such as MR-IVW (the mainly method), MR Egger, weighted median, and weighted mode. The results showed statistical significance with a P-value less than 0.05.

### Bayesian co-localization analysis

Bayesian colocalization analysis was used to assess the possibility of two features sharing the same causal variable. The “coloc” package (https://github.com/chr1swallace/coloc) was used to validate the argument [31]. Bayesian colocalization provided posterior probabilities for five hypotheses: hypothesis 0 (PPH0), hypothesis 1 (PPH1), hypothesis 2 (PPH2), hypothesis 3 (PPH3), and hypothesis 4 (PPH4). We validated the posterior probabilities of these hypotheses in the colocalization analysis of LDL-C with AP/NAFLD. SNPs were defined based on shared variants associated with the region. We defined a gene as having gene-based colocalization evidence with PPH4 > 80% determined by at least one algorithm [32, 33]. In this study, SNPs in the LDL-C instrumental variable were co-localized with acute pancreatitis/NAFLD one by one.

### Phenotype scanning

This study also conducted a phenotypic scan to search for associations between previously identified SNPs and other traits through genome-wide association studies. The phenotypic scan was performed using a “phenotype scanner” [34]. SNPs meeting the following criteria were considered pleiotropic: (i) they have significant genome-wide association (*P* < 5 × 10^–8^); (ii) the corresponding GWAS was conducted in European ancestry populations; (iii) these SNPs are associated with any known risk factors, such as gut microbiota, inflammatory cells, inflammatory proteins, low-density lipoprotein cholesterol, non-alcoholic fatty liver disease, and acute pancreatitis. Additionally, we also calculated the LD r^2^ values between prioritized SNPs to reveal potential connections.

### LDL-C: verification as a mediator between exposure and outcome

We validate AP and NAFLD using three exposures, respectively (Fig. 1). The filtering criterion of *P* < 5 * 10^–8^ was used to select instrumental variables (IVs) and select the same SNPs in both AP and NAFLD outcomes. Then, it conduct Mendelian randomization by IVW, MR-Egger, weighted median, and weighted mode analysis methods.

### MR internal validation of gut microbiota, inflammatory cells, and inflammatory related protein pqtl

To further validate LDL-C as a mediator and assess whether there is a direct causal relationship between exposure and outcome, this study conducted MR analysis using gut microbiota as exposure and acute pancreatitis/NAFLD as outcome. MR analysis of inflammatory cells as exposure and acute pancreatitis/NAFLD as outcome. The pqtl of inflammation related proteins was used as exposure, and MR analysis was performed on acute pancreatitis/NAFLD as outcome. The main analysis methods are IVW, MR-Egger, weighted median, and weighted mode.

### Data availability

The original study provides genome-wide summary statistics of exposure [28–30]. You can obtain the result data from the following website: https://gwas.mrcieu.ac.uk/. All data are publicly available and do not require ethical committee review.

## Results

### Bidirectional MR analysis of gut microbiota on LDL-C

**Fig. 2:**
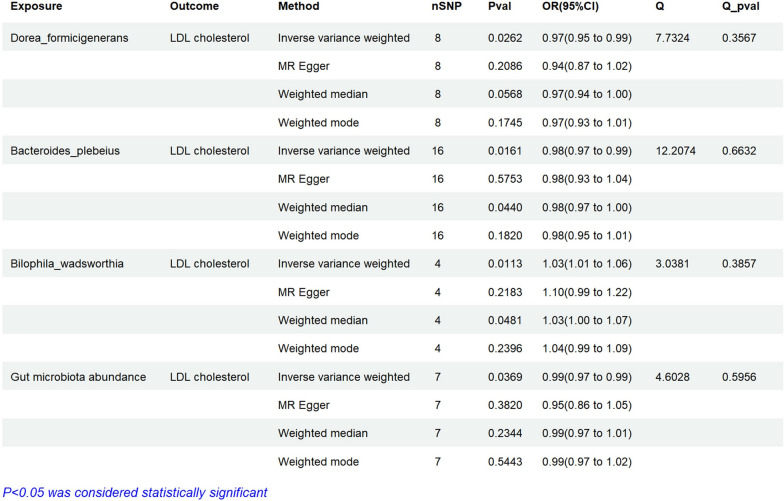
Assessing the causal relationships of Intestinal flora on the risk of LDL cholesterol. nSNP: Total number of instrumental variables used for analysis, OR > 1 Exposure increases risk of outcome. OR < 1 Exposure reduces the risk of the outcome. Q value: Heterogeneity analysis. Q Pvalue: P < 0.05, indicating heterogeneity

**Fig. 3:**
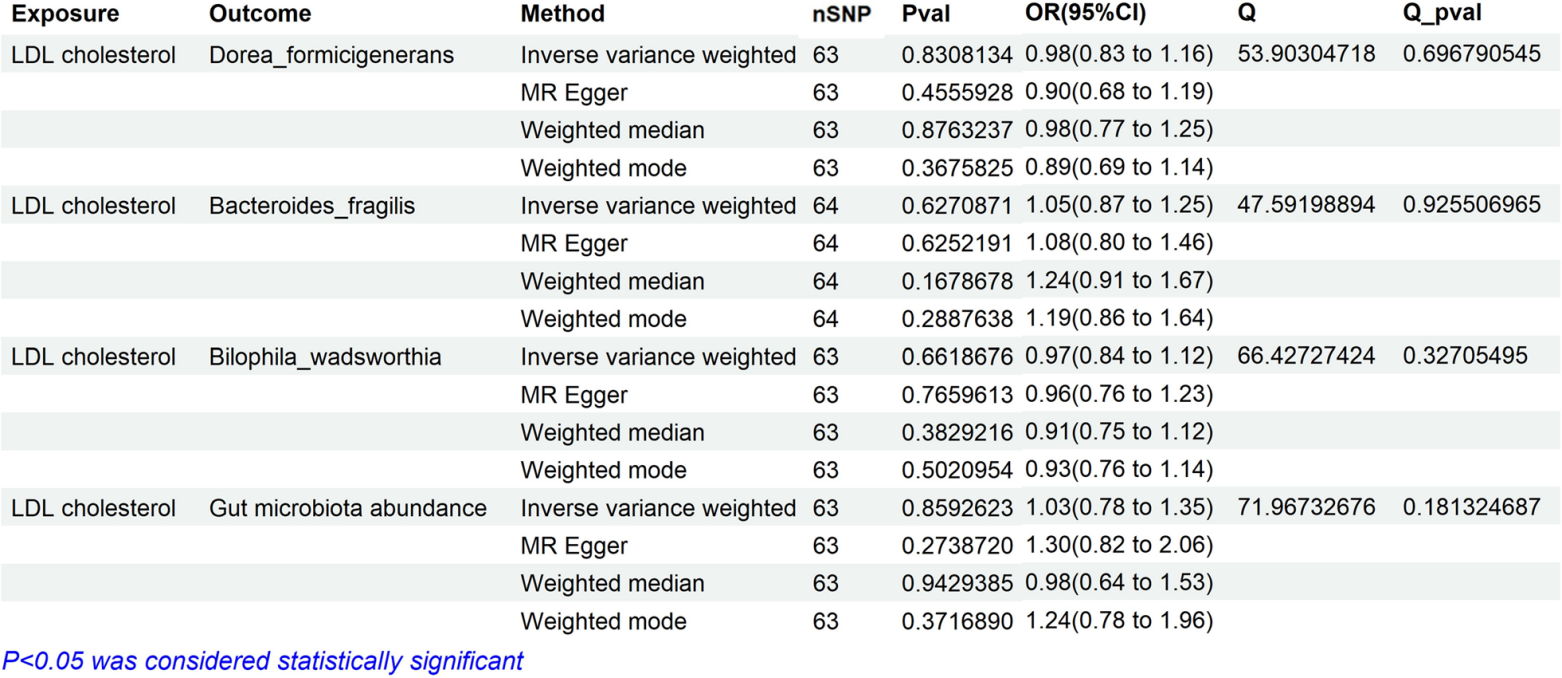
Reverse Mendelian randomization analysis of gut microbiota on the risk of LDL cholesterol

MR analysis was conducted on 412 gut microbiota using a P < 1 * 10–5 standard, with a total of 4 bacterial LDL-C pairs, including dorea formigenes, bacteria plebeius, biophila wadsworthia, and gut microbiota abundance (Fig. 2). Dorea formigenes (OR = 0.97, 95% CI 0.95–0.99, ***P = 0.026***), bacterial plebeius (OR = 0.98, 95% CI 0.97–0.99, ***P = 0.016***), and gut microbiota abundance (OR = 0.99, 95% CI 0.97–0.99, ***P = 0.0369***) can reduce the risk of LDL-C. However, the bacterial population of biophila wadsworthia (OR = 1.03, 95% CI 1.01–1.06, ***P = 0.011***) is at risk of increasing LDL-C (Fig. 2). No heterogeneity was detected for the gut microbiota analysed in the primary analysis (Fig. 2). To prevent reverse causality, we conducted a reverse MR analysis, and the results indicate that dorea formigenes (OR = 0.98, 95% CI 0.83–1.16, P = 0.83), biophila wadsworthia (OR = 0.97, 95% CI 0.84–1.12, P = 0.66), and gut microbiota abundance (OR = 1.03, 95% CI 0.78–1.35, P = 0.85). The bacterial plebeius was on found in the reverse MR analysis. Bacteria of the same genus dorea formigenerans (OR = 0.98, 95% CI 0.83–1.16, P = 0.83) has no statistical significance (Fig. 3). The P-values of heterogeneity analysis and pleiotropy analysis are all greater than 0.05(Figs. 2, 3).

### The causal relationship between inflammatory cells and LDL-C

**Fig. 4:**
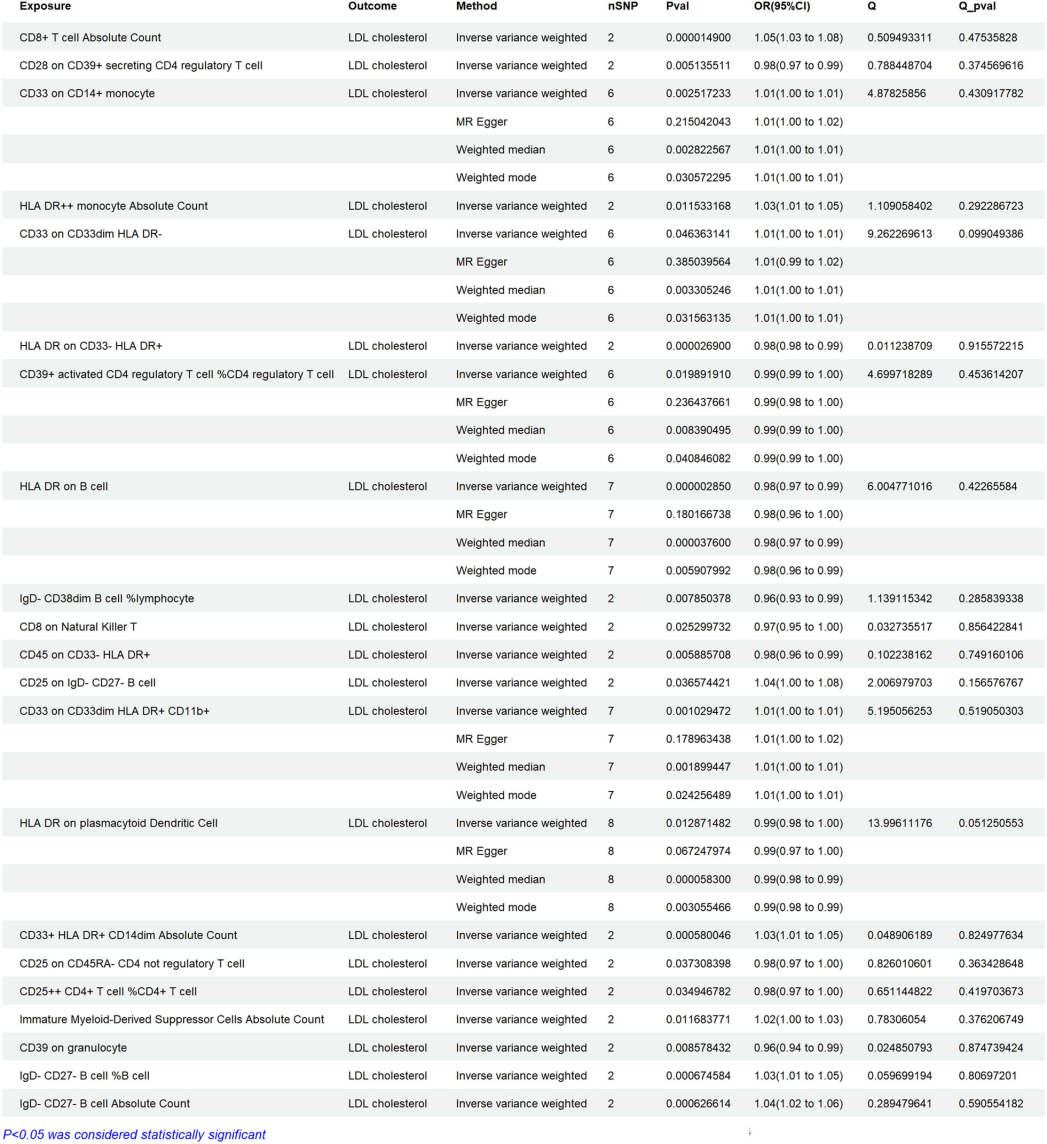
Assessing the causal relationships of Inflammatory cells on the risk of LDL cholesterol. nSNP: Total number of instrumental variables used for analysis, OR > 1 Exposure increases risk of outcome. OR < 1 Exposure reduces the risk of the outcome. Q value: Heterogeneity analysis. Q Pvalue: P < 0.05, indicating heterogeneity

**Fig. 5:**
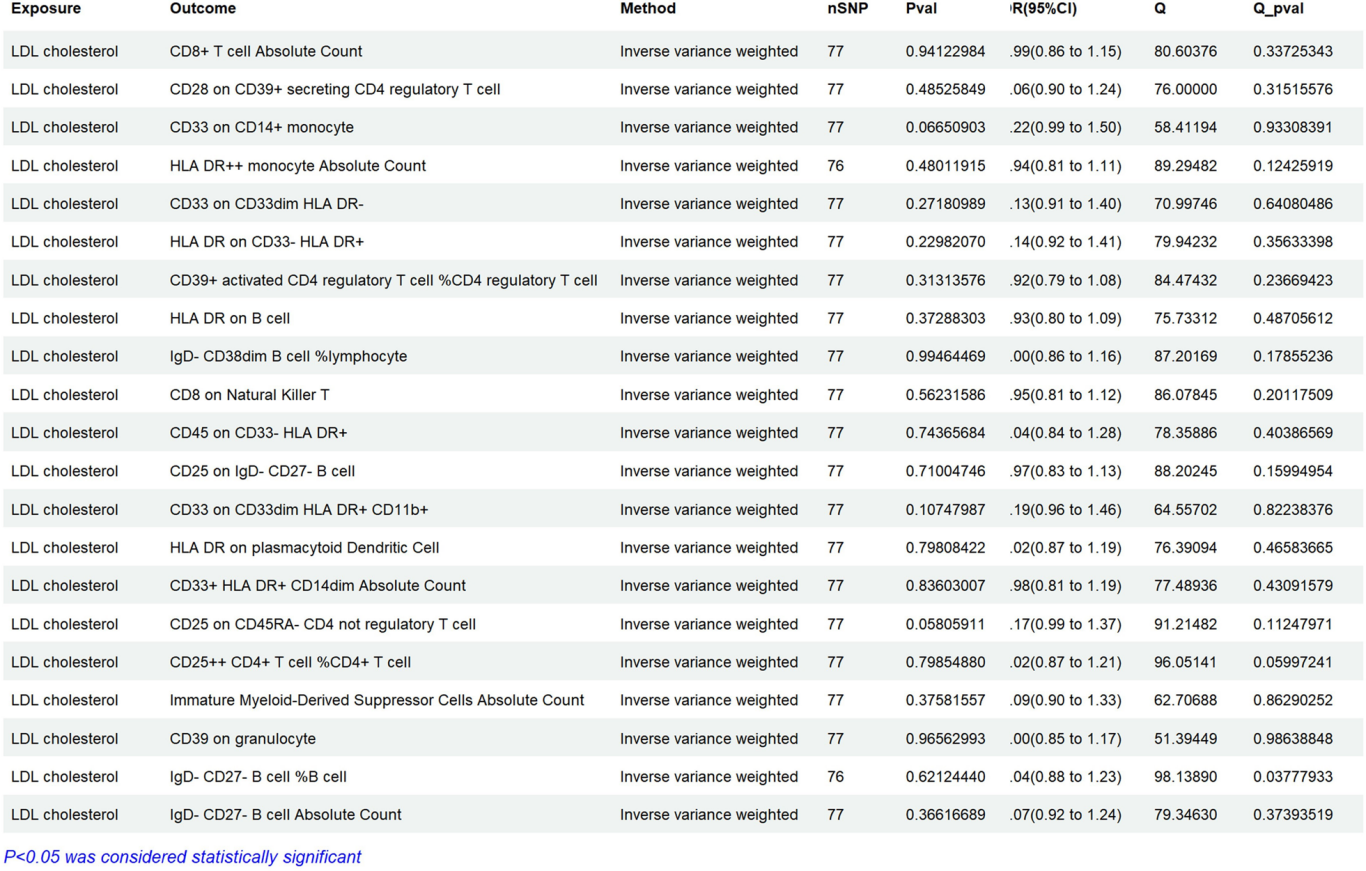
Reverse Mendelian randomization analysis of Inflammatory proteins on the risk of LDL cholesterol. nSNP: Total number of instrumental variables used for analysis, OR > 1 Exposure increases risk of outcome. OR < 1 Exposure reduces the risk of the outcome. Q value: Heterogeneity analysis. Q Pvalue: P < 0.05, indicating heterogeneity

The results of Mendelian randomization analysis showed that 10 types of cells are positively correlated with LDL-C,including CD8+ T cell Absolute Count (OR = 1.05, 95% CI 1.03–1.08, ***P < 0.01***), CD33 on CD14 + monocyte (OR = 1.01, 95% CI 1.00–1.02, ***P < 0.01***), HLA DR^++^ monocyte Absolute Count (OR = 1.03, 95% CI 1.01–1.05, ***P = 0.01***), CD33 on CD33dim HLA DR^−^ (OR = 1.01, 95% CI 1.00–1.01, ***P = 0.04***), CD25 on IgD^−^ CD27^e^ B cell (OR = 1.04, 95% CI 1.00–1.08, ***P = 0.036***), CD33 on CD33dim HLA DR^+^ CD11b+ (OR = 1.01, 95% CI 1.00–1.01, ***P < 0.01***), CD33^+^ HLA DR^+^ CD14dim Absolute Count (OR = 1.03, 95% CI 1.01–1.05, ***P < 0.01***), Immature Myeloid-Derived Suppressor Cells Absolute Count (OR = 1.02, 95% CI 1.00–1.03, ***P = 0.011***), IgD^−^ CD27− B cell %B cell (OR = 1.03, 95% CI 1.01–1.05, ***P < 0.01***), and IgD^−^ CD27^−^ B cell Absolute Count (OR = 1.04, 95% CI 1.02–1.06, ***P < 0.01***) may increase the risk of LDL-C. On the contrary, The results of Mendelian randomization analysis showed that 10 types of cells are negatively correlated with LDL-C, including CD28 on CD39^+^ secreting CD4 regulatory T cell (OR = 0.98, 95% CI 0.97–0.99, ***P = 0.005***), HLA DR on CD33^−^ HLA DR^+^(OR = 0.98, 95% CI, 0.98–0.99, ***P < 0.01***), CD39^e^ activated CD4 regulatory T cell %CD4 regulatory T cell (OR = 0.99, 95% CI 0.99–1.00, ***P = 0.019***), HLA DR on B cell (OR = 0.98, 95% CI 0.97–0.99, ***P < 0.01***), IgD^−^ CD38dim B cell %lymphocyte (OR = 0.96, 95% CI 0.93–0.99, ***P < 0.01***), CD8 on Natural Killer T (OR = 0.97, 95% CI 0.95–1.00, ***P = 0.025***), CD45 on CD33^−^ HLA DR^+^ (OR = 0.98, 95% CI 0.96–0.99, ***P < 0.01***), HLA DR on plasmacytoid Dendritic Cell (OR = 0.99, 95% CI 0.98–1.00, ***P = 0.012***), CD25 on CD45RA^−^ CD4 not regulatory T cell (OR = 0.98, 95% CI 0.97–1.00, ***P = 0.037***), CD25^++^ CD4^+^ T cell %CD4^+^ T cell (OR = 0.98, 95% CI 0.97–1.00, ***P = 0.034***), and CD39 on granulocyte (OR = 0.96, 95% CI 0.94–0.99, ***P < 0.01***, Fig. 4). In reverse MR analysis, we found that the* P*-value of MR-IVW in all inflammatory cells was greater than 0.05. The *P*-values of the heterogeneity in MR analysis of all inflammatory cells in Fig. 4 are greater than 0.05. The *P*-values of the pleiotropy in the Fig. 4 are Below 0.05. The IVW results of reverse Mendelian randomization analysis showed that there was no obvious reverse causal relationship between exposure and outcome (P > 0.05) (Fig. 5).

### The causal relationship between inflammatory proteins and LDL-C

**Fig. 6:**
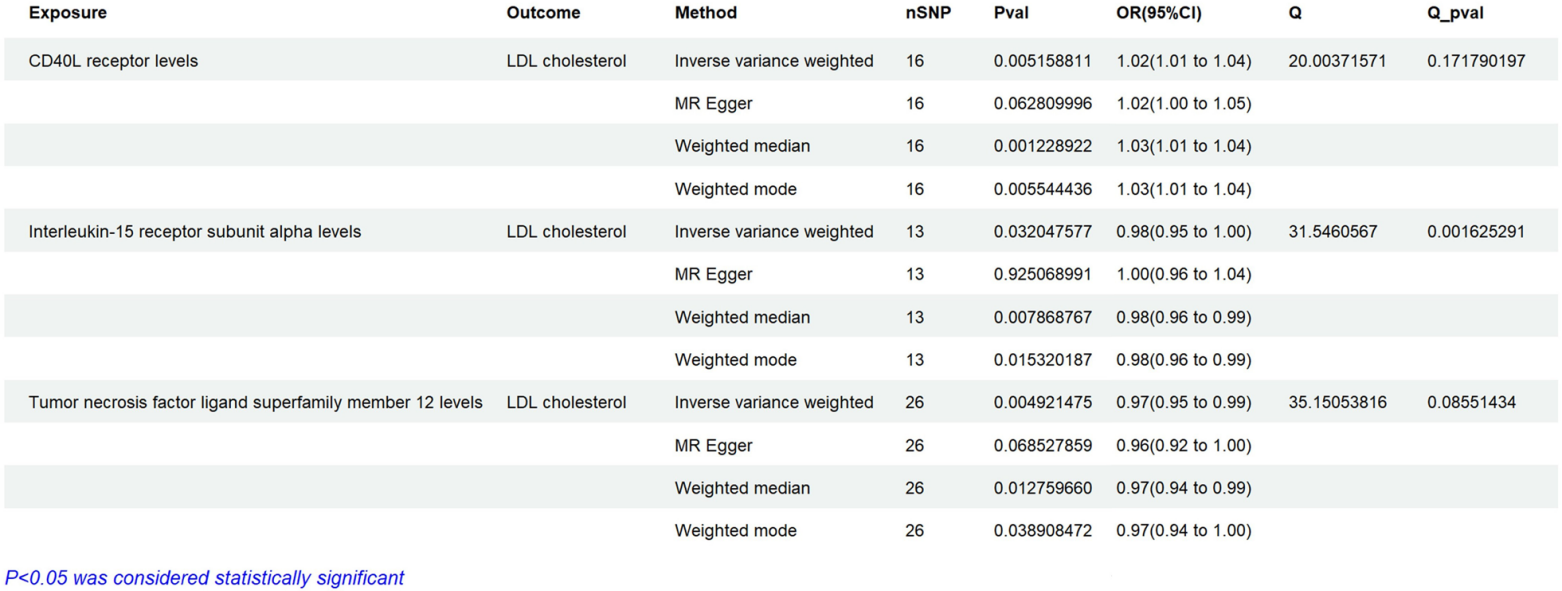
Assessing the causal relationships of Inflammatory cells on the risk of LDL cholesterol

Through MR analysis of pqtls of 91 inflammatory proteins, it was found that a total of 3 proteins were included, including CD40L receptor levels, Interleukin-15 receptor subunit alpha levels, and Tumor Necrosis factor ligand superfamily member 12 levels. High levels of CD40L receiver levels (OR = 1.02, 95% CI 1.01–1.04, ***P < 0.01***) may increase the risk of LDL-C (Fig. 6). On the contrary, high levels of Interleukin-15 receptor subunit alpha levels (OR = 0.98, 95% CI 0.95–1.00, ***P = 0.032***), and Tumor Necrosis factor ligand superfamily member 12 levels (OR = 0.98, 95% CI 0.95–1.00, ***P < 0.01***) may reduce the risk of LDL-C (Fig. 6). There was no statistically significant heterogeneity and pleiotropy in inflammatory proteins (Fig. 6). Summary-data-based Mendelian Randomization was also used to study the relationship between pqtl and LDL-C. The results of Additional file 1: Table S1 show that 7 SNPs including rs579459 have a causal relationship with LDL-C.

### The causal relationship between LDL-C and acute pancreatitis and non-alcoholic fatty liver disease

**Fig. 7:**
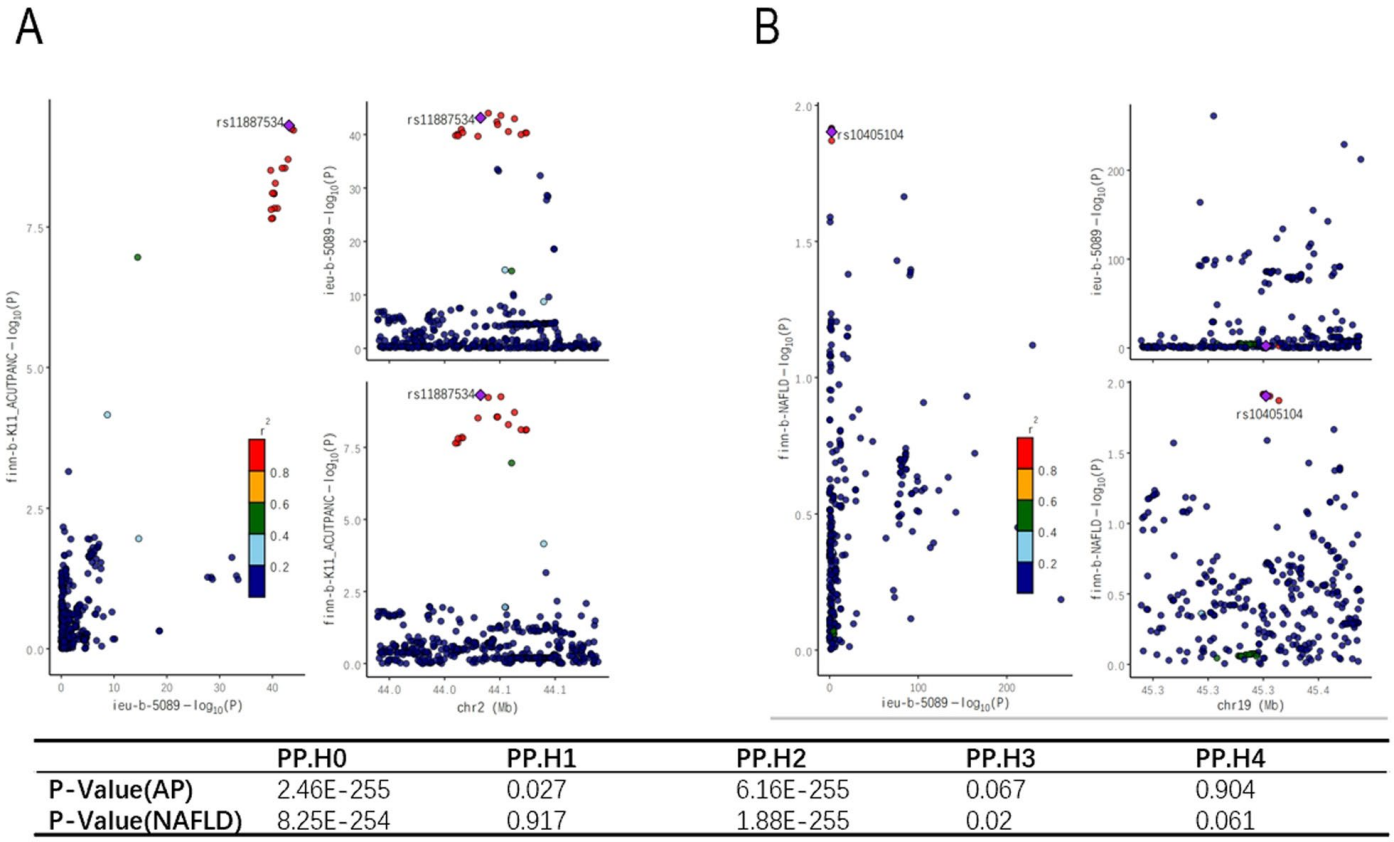
Co-location analysis, Co-localization results of LDL-C and acute pancreatitis (**A**), Co-localization results of LDL-C and NAFLD (**B**)

The causal relationship between LDL-C and acute pancreatitis (OR = 0.85, 95%CI 0.73–0.99, ***P = 0.04***). The results of MR-Egger (OR = 0.80, 95% CI 0.63–1.01, P = 0.06), Weighted medium (OR = 0.86, 95% CI 0.68–1.08, P = 0.2), and Weighted mode (OR = 0.84, 95% CI 0.68–1.08, P = 0.1) indicate that IVW-FE is reliable. The P-value of MR-PRESSO is equal to 0.11, indicating that LDL-C has no significant level pleiotropic effect on AP (Table 1). The reverse MR analysis of AP showed insufficient SNPs for analysis. The causal relationship between LDL-C and non-alcoholic fatty liver disease (OR = 0.71, 95%CI 0.54–0.94, ***P = 0.01***). The results of MR-Egger (OR = 0.66, 95% CI 0.42–1.03, P = 0.07), Weighted medium (OR = 0.66, 95% CI 0.44–0.98, ***P = 0.04***), and Weighted mode (OR = 0.69, 95% CI 0.49–0.97, ***P = 0.03***) indicate that IVW-FE is reliable. The P-value of MR-PRESSO is equal to 0.054, indicating that LDL-C has no significant level pleiotropic effect on NAFLD (Table 1). The reverse MR analysis of NAFLD showed insufficient SNPs for analysis. Co localization analysis showed a significant correlation between LDL-C and acute pancreatitis (Fig. 7A), that the P value of PP.H4 is 0.904, but the results of co localization analysis between LDL-C and NAFLD were negative (Fig. 7B), that the P value of PP.H4 is 0.061.

**Table 1:** Assessing the causal effects of LDL cholesterol on the risk of Nonalcoholic fatty liver disease and acute pancreatitis

Exposure	Outcome	Method	nSNP	Pval	OR	MRPresso-Pval
LDL cholesterol	Non-alcoholic fatty liver disease	Inverse variance weighted (fixed effects)	73	0.01	0.71 (0.54–0.94)	0.054
		MR Egger	73	0.07	0.66 (0.42–1.03)	
		Weighted median	73	0.04	0.66 (0.44–0.98)	
		Weighted mode	73	0.03	0.69 (0.49–0.97)	
LDL cholesterol	Acute pancreatitis	Inverse variance weighted (fixed effects)	74	0.04	0.85 (0.73–0.99)	0.11
		MR Egger	74	0.06	0.80 (0.63–1.01)	
		Weighted median	74	0.20	0.86 (0.68–1.08)	
		Weighted mode	74	0.10	0.84 (0.68–1.03)

### Verification of the causal relationship between gut microbiota, inflammatory cells, and inflammatory proteins (pqtls) and AP

In this MR analysis, the data in Additional file 1: Table S2 showed that in Fig. 2, all gut microbiota associated with LDL-C were unrelated (no corresponding SNP was found for the dorea formigenes genus, which was excluded for statistical purposes). The data in Additional file 1: Table S3 showed that in Fig. 4, all inflammatory cells associated with LDL-C were unrelated (Some inflammatory cells were not counted as they did not match the corresponding SNP). The data in Additional file 1: Table S4 showed that in Fig. 6, all inflammatory proteins associated with LDL-C were unrelated.

### Verification of the causal relationship between gut microbiota, inflammatory cells, and inflammatory proteins (pqtls) and NAFLD

In this MR analysis, the data in Additional file 1: Table S5 showed that in Fig. 2, all gut microbiota associated with LDL-C were unrelated (no corresponding SNP was found for the dorea formigenes genus, which was excluded for statistical purposes). The data in Additional file 1: Table S6 showed that in Fig. 4, all inflammatory cells associated with LDL-C were unrelated (Some inflammatory cells were not counted as they did not match the corresponding SNP). The data in Additional file 1: Table S7 showed that in Fig. 6, all inflammatory proteins associated with LDL-C were unrelated.

### The causal relationship between gut microbiota and AP

**Fig. 8:**
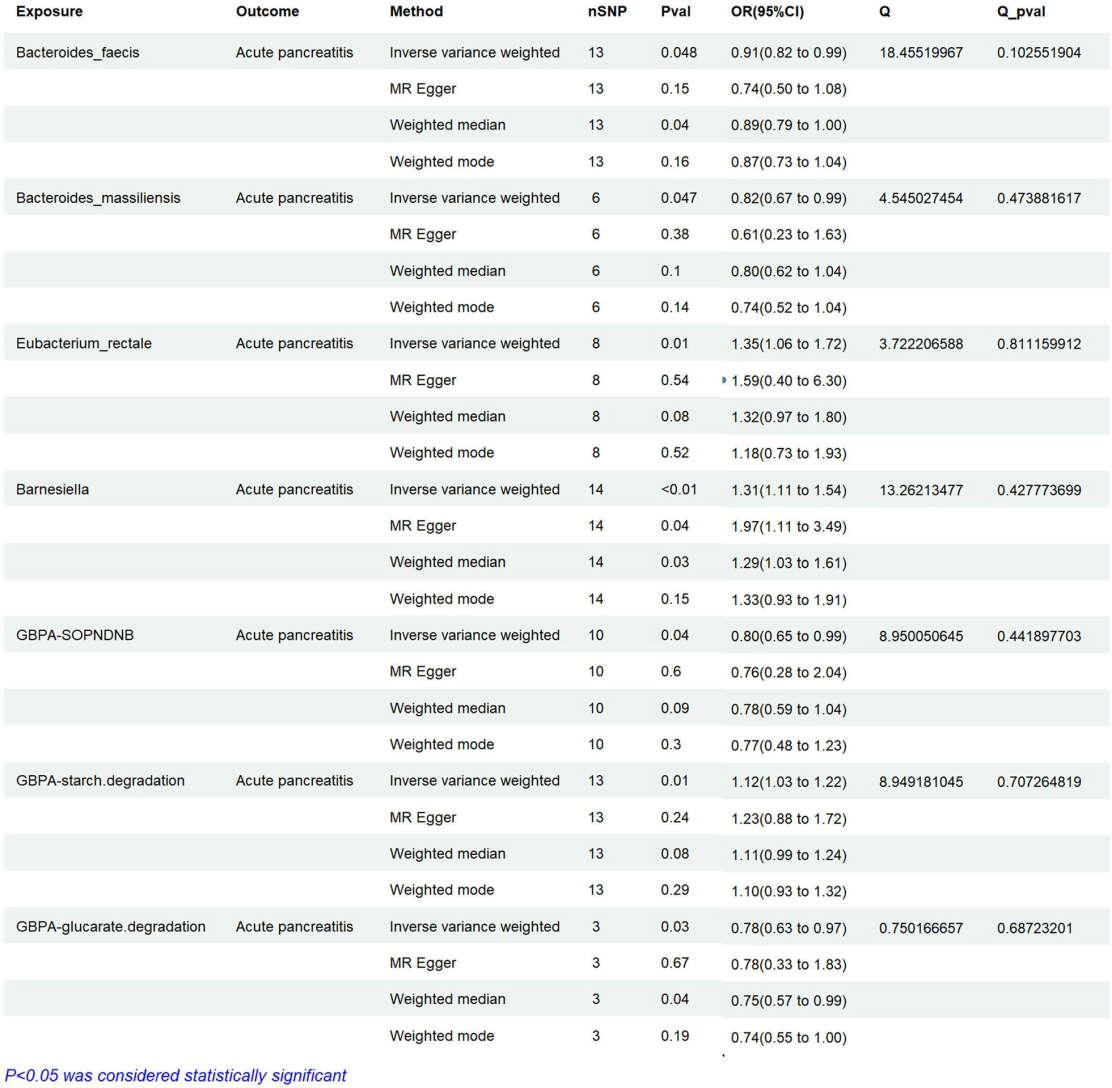
Assessing the causal relationships of Gut bacterial on the risk of AP. nSNP: Total number of instrumental variables used for analysis, OR > 1 Exposure increases risk of outcome. OR < 1 Exposure reduces the risk of the outcome. Q value: Heterogeneity analysis. Q Pvalue: P < 0.05, indicating heterogeneity

There is a total of 7 gut microbiota associated with acute pancreatitis, including bacteroides faecis, bacteroides massiliensis, eubacterium rectale, Barnesiella, gut bacterial pathway allowance in hyperpathway of purine nucleotides de novo biosynsis (GBPA-SOPNDNB), gut bacterial pathway abundance-starch degradation (GBPA-starch degradation), and gut bacterial pathway abundance-glucarate degradation (GBPA-glucarate degradation). Increasing the number of bacteroides faecis (OR = 0.91, 95%CI 0.82–0.99, ***P = 0.048***), bacteroides massiliensis (OR = 0.82, 95%CI 0.67–0.99, ***P = 0.047***), GBPA-SOPNDNB (OR = 0.80, 95%CI 0.65–0.99, ***P = 0.04***), GBPA-SOPNDNB (OR = 0.80, 95%CI 0.65–0.99, ***P = 0.04***), and GBPA-glucarate degradation (OR = 0.78, 95%CI 0.63–0.97, ***P = 0.03***) may reduce the risk of AP (Fig. 8). Increasing the number of eubacterium rectale (OR = 1.35, 95%CI 1.06–1.72, ***P = 0.01***), barnesiella(OR = 1.31, 95%CI 1.11–1.54, ***P < 0.01***), GBPA-SOPNDNB (OR = 0.80, 95%CI 0.65–0.99, ***P = 0.04***), and GBPA-starch degradation (OR = 1.12, 95%CI 1.03–1.22, ***P = 0.01***) may increase the risk of AP (Fig. 8). When using reverse MR analysis, it was found that AP had not enough SNPs to match the gut microbiota. This result suggests that acute pancreatitis may not affect the corresponding gut microbiota.

## Discussion

The pathogenesis of acute pancreatitis has undergone extensive investigations, leading to the acceptance of various established perspectives. These include impaired autophagy, premature activation of pancreatic protease, dysfunction in the unfolded protein response (UPR), mitochondrial dysfunction, endoplasmic reticulum stress, and aberrant calcium signaling [35–37]. Emerging studies have also implicated the influence of gut microbiota on the progression of acute pancreatitis. Specifically, the absence of TLR4 expression in intestinal epithelial cells exacerbates pancreatic and intestinal damage during acute pancreatitis. Dysbiosis of the gut microbiota, particularly the reduction in lactobacillus, appears to contribute significantly to this damage [38, 39]. Additionally, an association between cholesterol and acute pancreatitis has been observed. Cholesterol has the potential to induce inflammation by activating pathways such as TLR4, NLRP3, and NETs, indicating its vital role in the pathogenesis of acute pancreatitis [40]. Studies have highlighted the significance of pancreatic microvascular abnormalities and ischemia in the pathogenesis of acute pancreatitis [41]. Oxidized low-density lipoprotein has been implicated in disrupting coagulation system, promoting the formation of blood clot, and subsequently influencing organ blood supply [42]. Moreover, elevated levels of low-density lipoprotein cholesterol primarily worsen severe acute pancreatitis through mechanisms involving increased oxidative stress and amplified inflammatory response. This lipoprotein cholesterol also stimulates the generation of reactive oxygen species (ROS) while inhibiting nitric oxide synthesis [43]. Studies have unequivocally demonstrated the significant anti-inflammatory effects of nitric oxide. Nitric oxide exhibits the ability to inhibit the transcription factor NF-κB, a crucial regulator induced and produced by cytokines. This inhibitory action consequently reduces the release of inflammatory cytokines and mitigates mitochondrial ROS production [43]. The intricate interplay between ROS and NF-κB activation, coupled with lipid peroxidation, leads to the generation of inflammatory mediators such as IL-6 and monocyte chemoattractant protein 1 [44, 45].

These intricate mechanisms contribute significantly to the progressive development of acute pancreatitis. Certain studies have indicated that modified LDL-C has the potential to bind to receptors such as TLR2 and TLR4, thereby triggering the release of pro-inflammatory factors [46]. In the circulatory system, a substantial quantity of oxidized LDL-C exists, and when it combines with specific antibodies, it forms complexes that triggered inflammatory reactions involving macrophages and dendritic cells [47]. This study demonstrated a significant correlation between LDL-C and acute pancreatitis. Co-localization analysis further confirmed that rs11887534 can simultaneously influence LDL-C and the progression of acute pancreatitis, providing further evidence for a causal relationship between LDL-C and acute pancreatitis. The results of reverse Mendelian randomization analysis indicate that there is no significant causal relationship between AP and LDL-C. Dorea_formicigenerans, Bacteroides_plebeius, Bilophila_wadsworthia and the abundance of gut microbiota (Fig. 2) may affect acute pancreatitis by affecting LDL-C. These microbiotas have not been reported yet and may become targets for the treatment of AP. Reverse Mendelian randomization analysis demonstrated that there was no significant causal relationship between LDL-C and AP on gut microbiota. This study further validated the impact of these gut microbiota on AP by MR-analysis, and found that the P-value was greater than 0.05. Inflammatory cells and inflammatory proteins also have certain effects on LDL-C, inflammatory cells and inflammatory molecules not only directly impact the progression of AP, but also contribute to the symptoms and severity of AP through the LDL-C pathway. In short, this study discovered a novel mechanism in which gut microbiota, inflammatory cells, and inflammatory proteins affect acute pancreatitis through LDL-C as a mediator.

Non-alcoholic fatty liver disease stands as the primary cause of chronic liver disease in developed nations, with a consistently rise in its global prevalence [21]. This condition is close association with metabolic syndrome, characterized by central obesity, insulin resistance, hypertension, hyperlipidemia, and dyslipidemia [48]. Presently, the etiology of non-alcoholic fatty liver disease remains complex and not fully elucidated. The predominant theory among experts is the “multiple-hit” hypothesis, aims to explain its pathogenesis. According to this hypothesis, the interplay of factors such as gut microbiota, insulin resistance, and adipokines exerts profound influence on hepatic steatosis, oxidative stress, mitochondrial dysfunction, and inflammation in liver tissue [49]. Notably, the gut microbiota assumes significant relevance in non-alcoholic fatty liver disease and contributes by providing its components or metabolites. Inflammatory response triggered by bacterial endotoxins, peptidoglycans, DNA, and extracellular vesicles may hasten the onset of non-alcoholic fatty liver disease and the progression to non-alcoholic steatohepatitis (NASH) [50]. A multitude of gastrointestinal disorders, including nonalcoholic fatty liver disease, diabetes, and obesity, exhibit intricate associations with the gut microbiota [51, 52]. Evidence suggests that an imbalance in the gut microbiota can contribute to the development of nonalcoholic fatty liver disease [53–55]. Notably, a study has identified nonalcoholic fatty liver disease as an independent risk factor for LDL-C target levels [56]. However, a Mendelian randomization trial contradicts this notion by suggesting that NAFLD has limited impact on LDL-C. In contrast, the findings of a Mendelian randomization analysis demonstrate that LDL-C can potentially exacerbate the progression of NAFLD. Existing evidence indicates a connection between lipid metabolism and NAFLD, with LDL-C potentially assuming a pivotal role [57]. Another clinical study has established a correlation between LDL-C and NAFLD, suggesting an important involvement of LDL-C in this pathogenesis [58]. NAFLD manifests as a disease characterized by liver inflammation, fibrosis, and structural alterations [59]. Additionally, gut microbiota imbalance [60] and gut barrier dysfunction [61, 62] are believed to lead to an increase in bacterial translocation and the secretion of inflammatory cytokines and interferons [63], activating the inflammatory pathway in the liver. Therefore, this Mendelian randomization analysis effectively illuminated the impact of multi-omics on LDL-C and NAFLD by considering three levels of omics: gut microbiota, inflammatory cells, and inflammatory proteins. Unfortunately, the co-localization analysis does not definitively illustrate the relationship between LDL-C and NAFLD, likely due to the current incompleteness of available data.

## Conclusion

This study demonstrated the causal relationship between genomics and LDL-C through three omics, including gut microbiota, inflammatory cells, and inflammatory proteins. It also demonstrated the causal relationship between LDL-C and acute pancreatitis, as well as between LDL-C and NAFLD. These data indicate that these three omics can indirectly affect the progression of acute pancreatitis and NAFLD disease through LDL-C. It also indicates a causal relationship between seven gut microbiota and acute pancreatitis.

## Limitations

Our study has several limitations. Firstly, we only used data from one group of gut microbiotas to evaluate the impact of LDL-C, and did not validate our findings using data from others. Secondly, the data related to inflammatory cells and inflammatory proteins is relatively limited, and there is no other data to further validate our conclusion. Finally, add research and analysis between LDL-C and AP in the Nhance database to determine whether there is a correlation between LDL-C and AP. Establish a pancreatitis cell line through in vitro experiments, add LDL-C to the culture medium, and study Changes in inflammation-related pathways. Further establish a mouse pancreatitis animal model to clarify the role of LDL-C in the progression of inflammation in animals.

### Supplementary Information

**Additional file 1: Table S1.** Assessing the causal effects of gut microbiota on the risk of acute pancreatitis. **Table S2.** Assessing the causal effects of inflammatory cells on the risk of acute pancreatitis. **Table S3.** Assessing the causal effects of inflammatory proteins on the risk of acute pancreatitis. **Table S4.** Assessing the causal effects of gut microbiota on the risk of NAFLD. **Table S5.** Assessing the causal effects of inflammatory cells on the risk of NAFLD. **Table S6.** Assessing the causal effects of inflammatory proteins on the risk of NAFLD.

## Data Availability

The data of UKBiobank can be obtained in this website (https://pheweb.org/UKB-SAIGE/). The data for FinnGen can be obtained in this website (https://www.finngen.fi/en/access_results). The other websites resources: IEU OpenGWAS (https://gwas.mrcieu.ac.uk/), and Phenoscanner (http://www.phenoscanner.medschl.cam.ac.uk/).

## Abbreviations

P-qtls: Protein quantitative trait loci
NAFLD: Non-alcoholic fatty liver disease
LDL-C: Low-density lipoprotein cholesterol
AP: Acute pancreatitis
TLR2: Toll-like receptor 2
TLR4: Toll-like receptor 4
ROS: Reactive oxygen species
NF-κB: Nuclear factor kappa-B
UPR: Unfolded protein response
TLR4: Toll-like receptor 4
NLRP3: Nod-like receptor thermal protein domain associated protein 3
