# Supplementary material for: Exploring the interplay of gut microbiota, inflammation, and LDL-cholesterol: a multiomics Mendelian randomization analysis of their causal relationship in acute pancreatitis and non-alcoholic fatty liver disease
Source: J Transl Med. 2024 Feb 19;22:179. doi: 10.1186/s12967-024-04996-0 (PMC10875775; doi:10.1186/s12967-024-04996-0)
Supplement: Supplementary file 1 — Additional file 1: Table S1. Assessing the causal effects of gut microbiota on the risk of acute pancreatitis. Table S2. Assessing the causal effects of inflammatory cells on the risk of acute pancreatitis. Table S3. Assessing the causal effects of inflammatory proteins on the risk of acute pancreatitis. Table S4. Assessing the causal effects of gut microbiota on the risk of NAFLD. Table S5. Assessing the causal effects of inflammatory cells on the risk of NAFLD. Table S6. Assessing the causal effects of inflammatory proteins on the risk of NAFLD. [file 12967_2024_4996_MOESM1_ESM.docx]

**Table S1 Assessing the causal effects of gut microbiota on the risk of acute pancreatitis**

| **Exposure** | **Outcome** | **Method** | **nSNP** | **Pval** | **OR** |
| --- | --- | --- | --- | --- | --- |
| **Bacteroides_plebeius** | **Acute pancreatitis** | **Inverse variance weighted** | **7** | **0.45** | **1.04（0.92-1.18）** |
| **Bilophila_wadsworthia** | **Acute pancreatitis** | **Inverse variance weighted** | **7** | **0.53** | **1.08（0.83-1.42）** |
| **Gut microbiota abundance** | **Acute pancreatitis** | **Inverse variance weighted** | **7** | **0.88** | **0.99（0.88-1.11）** |

**Table S2** **Assessing the causal effects of inflammatory cells on the risk of acute pancreatitis**

| **Exposure** | **Outcome** | **Method** | **nSNP** | **Pval** | **OR** |
| --- | --- | --- | --- | --- | --- |
| **CD28 on CD39+ secreting CD4 regulatory T cell** | **Acute pancreatitis** | **Inverse variance weighted** | **2** | **0.06** | **1.12（0.99-1.27）** |
| **CD33 on CD14+ monocyte** | **Acute pancreatitis** | **Inverse variance weighted** | **5** | **0.5** | **0.99（0.94-1.03）** |
| **CD33 on CD33dim HLA DR-** | **Acute pancreatitis** | **Inverse variance weighted** | **6** | **0.96** | **1（0.96-1.04）** |
| **HLA DR on CD33- HLA DR+** | **Acute pancreatitis** | **Inverse variance weighted** | **2** | **0.34** | **0.97（0.9-1.04）** |
| **CD39+ activated CD4 regulatory T cell %CD4 regulatory T cell** | **Acute pancreatitis** | **Inverse variance weighted** | **6** | **0.33** | **1.04（0.96-1.11）** |
| **HLA DR on B cell** | **Acute pancreatitis** | **Inverse variance weighted** | **9** | **0.74** | **0.99（0.93-1.05）** |
| **IgD- CD38dim B cell %lymphocyte** | **Acute pancreatitis** | **Inverse variance weighted** | **2** | **0.36** | **0.88（0.66-1.07）** |
| **CD8 on Natural Killer T** | **Acute pancreatitis** | **Inverse variance weighted** | **2** | **0.09** | **1.21（0.97-1.51）** |
| **CD45 on CD33- HLA DR+** | **Acute pancreatitis** | **Inverse variance weighted** | **2** | **0.95** | **1（0.87-1.16）** |
| **CD25 on IgD- CD27- B cell** | **Acute pancreatitis** | **Inverse variance weighted** | **2** | **0.28** | **0.89（0.71-1.1）** |
| **CD33 on CD33dim HLA DR+ CD11b+** | **Acute pancreatitis** | **Inverse variance weighted** | **6** | **0.57** | **0.99（0.95-1.03）** |
| **HLA DR on plasmacytoid Dendritic Cell** | **Acute pancreatitis** | **Inverse variance weighted** | **8** | **0.45** | **0.98（0.93-1.03）** |
| **CD33+ HLA DR+ CD14dim Absolute Count** | **Acute pancreatitis** | **Inverse variance weighted** | **2** | **0.28** | **0.92（0.79-1.07）** |
| **CD25 on CD45RA- CD4 not regulatory T cell** | **Acute pancreatitis** | **Inverse variance weighted** | **2** | **0.21** | **0.88（0.72-1.07）** |
| **CD25++ CD4+ T cell %CD4+ T cell** | **Acute pancreatitis** | **Inverse variance weighted** | **2** | **0.17** | **0.87（0.71-1.06）** |
| **Immature Myeloid-Derived Suppressor Cells Absolute Count** | **Acute pancreatitis** | **Inverse variance weighted** | **2** | **0.58** | **0.97（0.86-1.09）** |
| **CD39 on granulocyte** | **Acute pancreatitis** | **Inverse variance weighted** | **2** | **0.26** | **1.19（0.88-1.61）** |
| **IgD- CD27- B cell %B cell** | **Acute pancreatitis** | **Inverse variance weighted** | **3** | **0.33** | **0.93（0.82-1.07）** |
| **IgD- CD27- B cell Absolute Count** | **Acute pancreatitis** | **Inverse variance weighted** | **2** | **0.58** | **0.95（0.79-1.14）** |

**Table S3 Assessing the causal effects of inflammatory proteins on the risk of acute pancreatitis**

| **Exposure** | **Outcome** | **Method** | **nSNP** | **Pval** | **OR** |
| --- | --- | --- | --- | --- | --- |
| **CD40L receptor levels** | **Acute pancreatitis** | **Inverse variance weighted** | **16** | **0.12** | **0.91（0.82-1.02）** |
| **Interleukin-15 receptor subunit alpha levels** | **Acute pancreatitis** | **Inverse variance weighted** | **13** | **0.26** | **1.07（0.94-1.21）** |
| **Tumor necrosis factor ligand superfamily member 12 levels** | **Acute pancreatitis** | **Inverse variance weighted** | **26** | **0.14** | **1.12（0.96-1.32)** |

**Table S4 Assessing the causal effects of gut microbiota on the risk of NAFLD**

| **Exposure** | **Outcome** | **Method** | **nSNP** | **Pval** | **OR** |
| --- | --- | --- | --- | --- | --- |
| **Bacteroides_plebeius** | **NAFLD** | **Inverse variance weighted** | **7** | **0.45** | **0.92(0.74-1.14)** |
| **Bilophila_wadsworthia** | **NAFLD** | **Inverse variance weighted** | **7** | **0.64** | **1.10(0.71-1.71)** |
| **Gut microbiota abundance** | **NAFLD** | **Inverse variance weighted** | **7** | **0.87** | **0.98(0,8-1.2)** |

**Table S5 Assessing the causal effects of inflammatory cells on the risk of NAFLD**

| **Exposure** | **Outcome** | **Method** | **nSNP** | **Pval** | **OR** |
| --- | --- | --- | --- | --- | --- |
| **CD28 on CD39+ secreting CD4 regulatory T cell** | **NAFLD** | **Inverse variance weighted** | **2** | **0.17** | **1.17(0.93-1.46)** |
| **CD33 on CD14+ monocyte** | **NAFLD** | **Inverse variance weighted** | **5** | **0.91** | **1(0.91-1.07)** |
| **CD33 on CD33dim HLA DR-** | **NAFLD** | **Inverse variance weighted** | **6** | **0.61** | **1.03(0.91-1.15)** |
| **HLA DR on CD33- HLA DR+** | **NAFLD** | **Inverse variance weighted** | **2** | **0.59** | **1.03(0.91-1.17)** |
| **CD39+ activated CD4 regulatory T cell %CD4 regulatory T cell** | **NAFLD** | **Inverse variance weighted (multiplicative random effects)** | **6** | **0.46** | **1.06(0.91-1.21)** |
| **HLA DR on B cell** | **NAFLD** | **Inverse variance weighted** | **9** | **0.47** | **0.96(0.85-1.07)** |
| **IgD- CD38dim B cell %lymphocyte** | **NAFLD** | **Inverse variance weighted** | **2** | **0.24** | **0.73(0.43-1.22)** |
| **CD8 on Natural Killer T** | **NAFLD** | **Inverse variance weighted** | **2** | **0.02** | **1.59(1.07-2.36)** |
| **CD45 on CD33- HLA DR+** | **NAFLD** | **Inverse variance weighted** | **2** | **0.73** | **0.96(0.76-1.21)** |
| **CD25 on IgD- CD27- B cell** | **NAFLD** | **Inverse variance weighted** | **2** | **0.06** | **1.47(0.98-2.17)** |
| **CD33 on CD33dim HLA DR+ CD11b+** | **NAFLD** | **Inverse variance weighted** | **6** | **0.77** | **0.99(0.91-1.06)** |
| **HLA DR on plasmacytoid Dendritic Cell** | **NAFLD** | **Inverse variance weighted** | **8** | **0.55** | **1.03(0.93-1.12)** |
| **CD33+ HLA DR+ CD14dim Absolute Count** | **NAFLD** | **Inverse variance weighted** | **2** | **0.73** | **0.93(0.63-1.37)** |
| **CD25 on CD45RA- CD4 not regulatory T cell** | **NAFLD** | **Inverse variance weighted** | **2** | **0.79** | **1.09(0.56-2.10)** |
| **CD25++ CD4+ T cell %CD4+ T cell** | **NAFLD** | **Inverse variance weighted** | **2** | **0.15** | **1.3(0.9-1.80)** |
| **Immature Myeloid-Derived Suppressor Cells Absolute Count** | **NAFLD** | **Inverse variance weighted** | **2** | **0.28** | **1.16(0.88-1.5)** |
| **CD39 on granulocyte** | **NAFLD** | **Inverse variance weighted** | **2** | **0.39** | **1.79(0.47-6.7)** |
| **IgD- CD27- B cell %B cell** | **NAFLD** | **Inverse variance weighted** | **3** | **0.06** | **1.27(0.99-1.63)** |
| **IgD- CD27- B cell Absolute Count** | **NAFLD** | **Inverse variance weighted** | **2** | **0.1** | **1.31(0.94-1.81)** |

**Table S6 Assessing the causal effects of inflammatory proteins on the risk of NAFLD**

| **Exposure** | **Outcome** | **Method** | **nSNP** | **Pval** | **OR** |
| --- | --- | --- | --- | --- | --- |
| **CD40L receptor levels** | **NAFLD** | **Inverse variance weighted** | **16** | **0.22** | **1.12（0.92-1.37）** |
| **Interleukin-15 receptor subunit alpha levels** | **NAFLD** | **Inverse variance weighted** | **13** | **0.84** | **0.97（0.78-1.21）** |
| **Tumor necrosis factor ligand superfamily member 12 levels** | **NAFLD** | **Inverse variance weighted** | **26** | **0.17** | **0.81（0.61-1.09）** |
